# An automatic analysis framework for FDOPA PET neuroimaging

**DOI:** 10.1177/0271678X231168687

**Published:** 2023-04-07

**Authors:** Giovanna Nordio, Rubaida Easmin, Alessio Giacomel, Ottavia Dipasquale, Daniel Martins, Steven Williams, Federico Turkheimer, Oliver Howes, Mattia Veronese, and the FDOPA PET imaging working group:, Sameer Jauhar, Maria Rogdaki, Robert McCutcheon, Stephen Kaar, Luke Vano, Grazia Rutigliano, Ilinca Angelescu, Faith Borgan, Enrico D’Ambrosio, Tarik Dahoun, Euitae Kim, Seoyoung Kim, Micheal Bloomfield, Alice Egerton, Arsime Demjaha, Ilaria Bonoldi, Chiara Nosarti, James Maccabe, Philip McGuire, Julian Matthews, Peter S Talbot

**Affiliations:** 1 Department of Neuroimaging, Institute of Psychiatry, Psychology & Neuroscience, King’s College London, London, UK; 2 Department of Psychosis Studies, Institute of Psychiatry, Psychology & Neuroscience, King’s College London, London, UK; 3 MRC London Institute of Medical Sciences, Hammersmith Hospital, London, UK; 4 Institute of Clinical Sciences (ICS), Faculty of Medicine, Imperial College London, UK; 5 Department of Information Engineering (DEI), University of Padua, Padua, Italy; 6 Psychiatric Imaging Group, MRC London Institute of Medical Sciences, Hammersmith Hospital, Imperial College London, London, UK; 7 Institute of Clinical Sciences, Faculty of Medicine, Imperial College, Imperial College London, London, UK; 8 South London and Maudsley NHS Foundation Trust, London, UK; 9 COMPASS Pathways plc, London, UK; 10 Psychiatric Neuroscience Group, Department of Basic Medical Sciences, Neuroscience and Sense Organs, University of Bari "Aldo Moro", Bari, Italy; 11 Department of Psychiatry, Warneford Hospital, University of Oxford, Oxford, UK; 12 Department of Psychiatry, Seoul National University Bundang Hospital, Gyeonggi-do, Republic of Korea; 13 Department of Psychiatry, College of Medicine, Seoul National University, Seoul, Republic of Korea; 14 Department of Brain & Cognitive Sciences, College of Natural Sciences, Seoul National University, Seoul, Republic of Korea; 15 Division of Psychiatry, Faculty of Brain Sciences, University College of London, London, UK; 16 Department of Child and Adolescent Psychiatry, Institute of Psychiatry, Psychology & Neurosicences, King’s College London, London, UK; 17 Centre for the Developing Brain, Division of Imaging Sciences & Biomedical Engineering, King's College London, London, UK; 18 Early Intervention Psychosis Clinical Academic Group, South London & Maudsley NHS Trust, London, UK; 19 Division of Neuroscience and Experimental Psychology, School of Biological Sciences, Faculty of Biology, Medicine and Health, University of Manchester, Manchester, UK

**Keywords:** FDOPA PET, dopamine synthesis, data analysis pipeline, neuroimaging biomarker, big data repository

## Abstract

In this study we evaluate the performance of a fully automated analytical framework for FDOPA PET neuroimaging data, and its sensitivity to demographic and experimental variables and processing parameters. An instance of XNAT imaging platform was used to store the King’s College London institutional brain FDOPA PET imaging archive, alongside individual demographics and clinical information. By re-engineering the historical Matlab-based scripts for FDOPA PET analysis, a fully automated analysis pipeline for imaging processing and data quantification was implemented in Python and integrated in XNAT. The final data repository includes 892 FDOPA PET scans organized from 23 different studies. We found good reproducibility of the data analysis by the automated pipeline (in the striatum for the Ki^cer^: for the controls ICC = 0.71, for the psychotic patients ICC = 0.88). From the demographic and experimental variables assessed, gender was found to most influence striatal dopamine synthesis capacity (F = 10.7, p < 0.001), with women showing greater dopamine synthesis capacity than men. Our automated analysis pipeline represents a valid resourse for standardised and robust quantification of dopamine synthesis capacity using FDOPA PET data. Combining information from different neuroimaging studies has allowed us to test it comprehensively and to validate its replicability and reproducibility performances on a large sample size.

## Introduction

**Figure 1.**
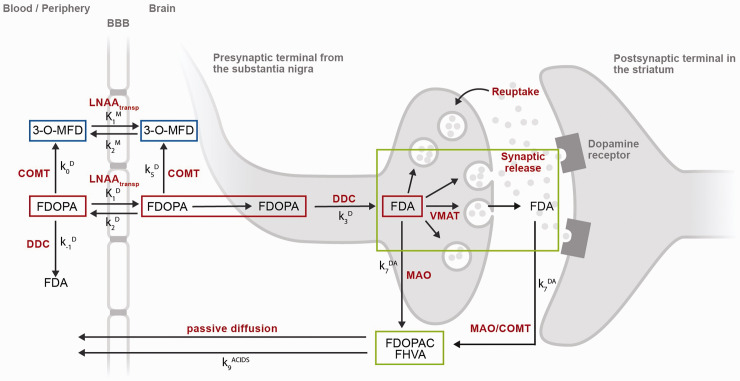
[^18^F]Fluorodopa (FDOPA) PET tracer kinetics in brain and periphery. After intravenous injection, the FDOPA in circulation is O-methylated at apparent rate constant k_0_^D^ (min^−1^) by cathecol-O-methyltransferase (COMT) to form 3-O-methyl-fluorodopa (3-O-MFD). Alternatively, the FDOPA in circulation can be decarboxylated at apparent rate constant k_-1_^D^ (min^−1^) by the enzyme DOPA decarboxylase (DDC) to form [^18^F]fluorodopamine (FDA). Both FDOPA and its COMT metabolite are subsequently cleared from circulation by renal elimination or reversibly transferred across the blood-brain-barrier by the common carrier of large neutral amino acids (LNAA_transp_). This reversible plasma-to-brain transport is defined through the unidirectional blood-brain clearances of FDOPA (K_1_^D^; mL/g min) and 3-O-MFD (K_1_^M^; mL/g min), and the corresponding rate constants for clearance back to circulation (k_2_^D^, k_2_^M^; min^−1^). FDOPA in brain tissue can be O-methylated at apparent rate constant k_5_^D^ (min^−1^) by COMT or decarboxylated at the rate constant k_3_^D^ (min^−1^) to form FDA. FDA is reversibly sequestered in vesicles by the vesicular monoamine transporter (VMAT) and then released into the synaptic cleft as part of both tonic and phasic dopamine release. FDA can then be reabsorbed into the presynaptic terminal and possibly be restored or metabolized by monoamine oxidase (MAO). Cytosolic FDA can also diffuse away from its source neuron to undergo metabolic destruction at another site; or it can be decomposed by MAO at rate constant k_7_^DA^ (min^−1^), yielding the acid metabolites [^18^F]fluorodihydroxyphenylacetic acid (FDOPAC) and [^18^F]fluorohomovanillic acid (FHVA). The acidic metabolites of FDA are together eliminated from brain by passive diffusion at rate constant k_9_^ACIDS^ (min^−1^). FDOPA PET signal in striatum might require 3 different levels of modelling: a compartmental model representing FDOPA accumulation in brain (red compartments), a compartmental model representing the exchange between blood and brain of the FDOPA metabolite 3-O-MFD (blue compartments), and a compartmental model representing the clearance of [^18^F]fluorodopamine (FDA) and its acidic metabolites FDOPAC and FHVA (green compartments). None of these models include the coefficient of brain tissue methylation of FDopa k_5_^D^, because it is assumed to be negligible throughout the brain.

Positron emission tomography (PET), in combination with the 6-[18F]fluoro-L-dopa (FDOPA) radiolabelled tracer, has been extensively used to image the dopamine system in vivo in living human brain.^1^ Accumulation of FDOPA in the brain parenchyma reflects its transport, decarboxylation into labelled dopamine, and vesicular uptake in the nigrostriatal presynaptic nerve terminals (Figure 1). The tracer was introduced in 1983^2^ to quantify the integrity of the nigrostriatal dopamine and it found immediate application in subclinical models of dopamine neuronal damage and in Parkinson’s Disease (PD) studies.^3,4^ However, it took 39 years before it received FDA approval as an imaging agent to visualize dopaminergic nerve terminals in the striatum of patients with suspected Parkinsonian syndromes.^5^ In neuro-oncology, the use of FDOPA PET and F-fluoroethyl-L-tyrosine (FET) PET tracers have shown higher uptake in neuroplastic tissue and relatively low uptake in normal brain when compared to 18 F-fluoro-deoxy-glucose (FDG).^6,7^ Particularly, FDOPA PET has been used as an amino-acid tracer to detect both primary and recurrent gliomas, outperforming standard FDG PET integrated with computed tomography (^18^F-FDG PET/CT) in terms of both accuracy and sensitivity for differentiating high-grade from low-grade gliomas.^8,9^ In psychiatry, FDOPA PET has been extensively used to quantify the dopamine system in the pathophysiology of psychotic and other symptoms across conditions, including schizophrenia,^10,11^ bipolar disorder,^12^ 22q11 syndrome,^13^ attention deficit disorders (ADHD),^14^ and substance dependence.^15^ Several lines of evidence have linked FDOPA PET to treatment response in psychosis^16–19^ suggesting that it might be used as a neurochemical basis to discriminate between patients likely to respond and those unlikely to respond to first-line antipsychotic drugs.^20^

The use of FDOPA PET in discriminating response in psychsosis or as diagnostic biomarker for oncology would require further validation on larger clinical datasets, aiming to support future individualized treatment and patient stratification across the different brain diseases. However, to reach clinical translation, FDOPA PET would also require a suitable data infrastructure and robust analytical protocols to ensure high quality of the data, accurate quantification, and replicable results. As for any modern neuroimaging biomarker, the inhability to provide sufficient companion data and the lack of a reproducible analytical framework would hamper FDOPA PET applicability.^21,22^ An additional obstacle in the creation of such infrastructure is the inconsistency and variety in neuroimaging data and file format, which has a direct impact on the quality and confidence of the data. The creation of large neuroimaging repositories that gather data from multiple sites and sources inevitably faces the problem of data harmonization,^23^ and with the current fragmentation, it is very difficult (if not impossible) to create a single data management and analysis system that works for all the possible scenarios.

The reproducibility of the data analysis is another requirement for any clinical translation of a neuroimaging biomarker to be effective. Poor scientific reproducibility is in fact embedded in the complexity of the data as well as in their analysis pipelines, making difficult to guarantee transparent and certified analytical processes.^24^ For example, it is well-known that the neuroimaging results can be highly dependent on the analytical method chosen. A recent study, in which 70 independent teams were asked to analyse the same MRI dataset, led to significant discrepancies between execution and results, and demonstrates how the flexibility of the analytical approaches leads to important differences in the quantification of the data.^25^ Similar findings were also observed for PET neuroimaging.^26^ In the case of FDOPA PET, there are several different analytical methods available, and ensuring reproducibility between these methods is far from trivial.^27^ Kinetic modelling and imaging pre-processing methods are necessary steps to isolate the biological components of interest from the measured FDOPA PET signal (Figure 1). Furthermore, quantification of FDOPA PET imaging consists of the measurement of the activity of aromatic amino acid decarboxylase for dopamine production, which returns information about the functional integrity of the presynaptic dopaminergic synthesis.^27^ This information of interest needs to be isolated from the total measured PET radioactivity, removing the contribution of the tracer metabolism and non-specific binding.^27^ It follows that the statistical proprieties of the radioligand in term of reproducibility and biological variability are not sufficient to guarantee the applicability of the method in clinical setting, and both the tracer kinetic modelling and the imaging data analysis pipeline need to be validated as constitutive parts of the FDOPA PET imaging biomarker.

This study aims to present and validate a new infrastructure for automated analysis of FDOPA PET neuroimaging, designed with the aspiration to provide a robust benchmark for the analysis of PET data acquired with this radiotracer, facilitating FDOPA PET imaging implemetation across sites and research institutions. The project takes advantage of a large FDOPA PET data repository available at the Institute of Psychiatry Psychology and Neuroscience (IoPPN) at King’s College London that has been exploited to test analysis pipeline replicability and reproducibility, as well as its sensitivity to processing parameters and experimental and demographic covariates.

## Methods

### FDOPA PET data acquisition

All FDOPA PET imaging sessions in the database were acquired with a continuous dynamic acquisition (no blood sampling), with scanning beginning with the tracer injection and lasting for 90-95 minutes. During this time, the participant was required to lie still in the PET scanner, with head rests to limit subject head motion. All participants received carbidopa (150 mg) and entacapone (400 mg) orally ∼1 hour before imaging. Both drugs are used to increase the signal-to-noise (SNR) of the tracer uptake in brain tissue by reducing the peripheral formation of radiolabelled dopamine and the 3-O-methyl-[^18^F]fluorodopa brain-penetrating metabolite, respectively.^20^ The FDOPA tracer (injected dose ranging from 86.4 to 414.4 MBq,) was administered by intravenous bolus injection after the acquisition of a brain CT or MRI for attenuation correction, depending on the scanner availability at each imaging site. PET data reconstruction varied across imaging sites and scanner types, but all included correction for random noise, scatter and tissue attenuation.

All the research protocols for data acquisitions were approved by local ethics committees and institutional revision boards including the Institute of Psychiatry, King’s College, London, England, research ethics committee; the South London and Maudsley/Institute of Psychiatry NHS Trust, London-West London & GTAC Research Ethics Committee; the Administration of Radioactive Substances Advisory Committee (ARSAC); the Hammersmith Research Ethics Committee; the East of England-Cambridge East NHS Research Ethics Committee; Seoul National University Hospital, Seoul, Korea. Full details on approval protocol numbers are reported on.^12,13,15,18–20,28–31^ Informed written consent was obtained for all the participants and the studies were conducted following the Declaration of Helsinki and Good Clinical Practice.

### Data management infrastructure

Our data management infrastructure was built using XNAT imaging technology.^32^ A bespoken installation of XNAT platform was deployed using the Neuroimaging Analysis Network at the Centre for Neuroimaging Sciences (King’s College London) to store for each subject’s demographic, clinical information and FDOPA PET imaging data. Representational State Transfer (REST) Application-Program Interface (API) was used to upload data.

For each subject, a scan imaging session was defined by a minimum amount of data which included dynamic FDOPA PET images (both attenuation-corrected and not attenuation-corrected), together with an ancillary file containing information regarding radiochemistry, date and time of scanning, timing of the acquisition and dynamic framing. Prior to storage in XNAT, the data were anonymized, harmonized in neurological convention, and corrected for radioisotope decay to ensure consistency across the data (Supplementary Figure 1). These features are quite standard in modern neuroimaging PET scanners, but they were not the standard for some of the oldest FDOPA PET scans included in the historical archive.

XNAT includes the Container Service plugin, which permits processes to be run on the stored data via REST API, using the processing utilities available in Docker containers.^33^ Taking advantage of this feature, analytical pipelines can be integrated in XNAT, allowing their automatic execution directly on the stored data and the storage of the outcomes at scan level (Supplementary Figure 1). An overview of the all the software tools included in the platform is reported in Supplementary Table 1.

### Automated analysis pipeline

The analysis pipeline was developed to align with our previous FDOPA imaging studies published by the Psychiatric Imaging Group (King’s College London) over the last two decades.^28–31,34–36^ In all these studies, the analysis of FDOPA PET imaging data was performed in MATLAB (Mathworks) and organised using a set of in-house scripts, which were manually executed and quality-controlled for each individual scan.

The overall process of FDOPA PET data analysis can be described as follows. First, dynamically non-attenuated and attenuated FDOPA PET images are inputted into the pipeline. The non-attenuated dynamic images are motion corrected frame-to-frame to a single reference frame with a linear transformation using Statistical Parametric Mapping (SPM) realign function.^37^ The reference frame is chosen at 15 min, as it represents an optimum trade-off between signal-to-noise ratio and radiotracer activity across all the brain tissues for a bolus injection FDOPA brain PET imaging scan (Supplementary Figure 2). The motion information is then used to realign the attenuated dynamic images, which are then summed together to create a motion-corrected individual static PET image. The information extrapolated during the motion correction step is used to quality control the data. This includes the “total motion”, estimated from geometrical realignment (i.e. combination of the translations in x, y and z directions and rotations in pitch, yaw and roll) of the individual PET frames, and the “number of spikes”, calculated as number of between-frames realignments exceeding a predefined threshold (5 mm, corresponding to the minimal spatial resolution detectable with standard clinical PET scanners^38^) If one or more spikes are detected, the scan is flagged with potential motion artifacts as this could indicate a potential miss-alignment with scan attenuation correction map (eigher CT-based or MR-based). A tracer-specific template and atlas defining the striatum and cerebellum are co-registered to each individual motion-correct static PET image, using SPM 12 (https://www.fil.ion.ucl.ac.uk/spm/). To segment both the basal ganglia (main area of activity for FDOPA PET) and whole brain, two different atlases are used to extract the radiotracer activity at region of interest level: 1) an in-house Montreal Neurological Institute (MNI)-based atlas including the whole striatum and its limbic, associative, and sensorimotor functional subdivisions as defined by Martinez et al;^39^ 2) the adult maximum probability brain atlas developed by Hammers et al to quantify extrastriatal regions.^40^ An overview of these regions is reported in Supplementary Figure 3. The Gjedde-Patlak Graphical approach,^41,42^ with the cerebellum as reference region extracted as defined in the Martinez atlas,^39^ is applied region-wise and voxel-wise to quantify Ki^cer^ (unit 1/min), a kinetic parameter used as a proxy of dopamine synthesis capacity.^27^ The t* for this analysis was fixed to 20 minutes, accordingly with previous literature.^12^ Prior to calculation of the Ki^cer^ parametric images, the images are denoised using a Chambolle Total Variation^43^ method. The parametric image for each scan is finally normalised into MNI standard coordinates using the participant’s PET summation image to calculate the image transformation field (non linear transformation). The FDOPA quantification is performed at individual space in order to avoid any alteration that might have been introduced with the normalization. The Standardized Uptake Value Ratio (SUVr) is also calculated as ratio of the tracer activity to that in the reference region (i.e. mean cerebellar FDOPA PET activity). The interval 60–75 min after the injection of the radiotracer is used for this analysis, since in this time window the Gjedde-Patlak plot is linear and used to derive Ki^cer^.^20,27^

Starting from the available MATLAB code, a fully automated version of this analysis pipeline was written in Python using its standard libraries (NumPy, SciPy, NiBabel, etc.), and integrated in XNAT. Specifically, SPM functions were implemented using the Nipype SPM interfaces, while for image denoising the Chambolle Total Variation function from the scikit-image package was used. Image quantification with Patlak analysis was rewritten starting from the mathematical formulation. An additional set of functions was integrated with the system to extract demographic, clinical, and analytical information in a summary report associated to each scan, automatically created, and stored in XNAT.

### Validation of the automated data analysis pipeline for FDOPA PET quantification

#### Comparison between automated and manual results

All the historical FDOPA scans were automatically re-analysed using the XNAT-based pipeline. A total sample of 521 scans was used to evaluate agreement between XNAT-based and the already available MATLAB-based results, using the same analysis settings.

#### Asssessment of test and retest reproducibility

Data from two independent different datasets were used to test XNAT-based pipeline reproducibility.

The first dataset comprised FDOPA PET test-retest imaging data from 7 healthy controls, dynamically acquired using an ECAT/EXACT3D: Siemens/CTI (Knoxville, Tennessee) PET tomograph. Approximately 150 MBq of 18 F-DOPA was administered by bolus intravenous injection 30 seconds after the start of the PET imaging. Data were acquired in emission mode for 95 minutes, for a total of 26 time-frames reconstructed using a 3-dimensional reprojection algorithm. Full details of the research protocol and subject inclusion criteria are reported in the original reference.^36^

The second dataset comprised FDOPA PET imaging data from 7 patients with psychosis before and after placebo, dynamically acquired using a Siemens Hi-Rez Biograph 6 PET scanner (Siemens, Erlangen, Germany) in 3 D mode. Approximately 150 MBq of 18 F-DOPA was administered by bolus intravenous injection after acquiring a CT scan for attenuation correction. PET data were acquired in 32 frames of increasing duration over the 95 min scan (frame intervals: 8 × 15 s, 3 × 60 s, 5 × 120 s, 16 × 300 s).

#### Impact of motion-correction and atlas coregistration to kinetic modelling

The effect of the detected number of spikes during motion correction on the FDOPA quantification was evaluated on all the data stored in XNAT, using a linear mixed model implemented with Jamovi (Version 2.0).

The effect of using the reliagned summed PET (PETtoPET) or individual structural MRI data (MRItoPET) for the atlas coregistration was investigated on the patient test-retest dataset. The sensitivity of dopamine synthesis capacity estimates and their reproducibility to the atlas coregistration method were evaluated in the striatal region and its subdivsions, as well as in extra-striatal regions (globus pallidus, substantia nigra, thalamus, hippocampus, amygdala, anterior cingulate cortex) which are known to be involved in the dopamine pathways.

### Identification of experimental and demographical covariates for FDOPA PET imaging

To investigate the presence of potential confounding factors to account in statistical analysis of FDOPA PET imaging data, the baseline scans (no pre-scan intervention) of healthy controls were selected from the database together with their experimental and demographic information. The final sample included 115 scans acquired from 3 different PET tomographs (Siemens Biograph 6 Hi-Rez, Siemens Biograph 6TruePoint, ECAT/EXACT3D) with an injected radioactivity below 200 MBq and acquisition time of 95 minutes. The experimental variables included tomograph type and radiochemistry measures (i.e. injected radioactivity, injected cold mass, specific activity) and the demographic variables included participant gender, age and weight at the time of scanning. A similar analysis was then repeated on a subsample (N = 103 scans), where the data were acquired using the Biograph PET tomographs (Siemens Biograph 6 Hi-Rez, Siemens Biograph 6 TruePoint) only.

### Statistical methods

For statistical analysis, GraphPad Prism v9 for Mac (GraphPad Software, La Jolla, CA) and SPSS (Version 27) were used. The Ki^cer^ estimates obtained from the XNAT and MATLAB analysis pipelines were compared using the Bland-Altman plot with 95% limits of agreement.^44^ Correlation and mean absolute percentage difference between the two pipelines were also calculated for the following six regions of interest (ROIs): whole striatum, right striatum, left striatum, whole sensorimotor subdivision, whole limbic subdivision, and whole associative subdivision. These ROIs are commonly the primary areas of analysis in PET imaging dopamine studies.^39,45^ For both XNAT and MATLAB pipelines, test-retest reliability was estimated with the Intraclass Correlation coefficient (ICC) using a 2-way mixed-model in SPSS (version 27, IBM®), while the within-subject variation was calculated as the absolute percentage test-retest difference.^36^ A p-value <0.05 was considered statistically significant. Similar statistical methods were also used to evaluate the effect of atlas coregisgtration on the reproducibility of the FDOPA quantification.

To investigate the effect of demographic and experimental variables on dopamine measures, a multi-linear regression model analysis was run with the K_i_^cer^ and SUVr of the whole striatum as dependent variable and both experimental and demographic variables as covariates.

## Results

### Database

The final infrastructure included 892 FDOPA PET scans from 23 different studies. Both primary and secondary data were organized following the same structure and naming convention, which facilitates data management and ensures homogeneity across the database. After removing commercials studies for which we did not have permission for data reuse, the infrastructure consisted of 792 FDOPA PET scans from 666 individuals (female 33.9%, healthy controls 29.1%) collected from four different imaging sites between 2004–2021 (Table 1). The mean age of the participants was 28.7 years (*range = 18–65, S.D. = 8.5*) with a mean weight of 75.9 kg (*range = 38–136, S.D. = 17.3*). All scans were acquired from five separate tomographs (*Siemens Biograph 6 Hi-Rez, Siemens Biograph 40 TruePoint, Siemens Biograph 6 TruePoint, ECAT/EXACT3D, GE SIGNA PET/MR*) with a mean injected radioactivity of 188.2 MBq (*range = 86.4–414.4, S.D. = 80.1*).

**Table 1.** Demographic, experimental and imaging site information of the data (excluding the commercial studies) stored in XNAT.

Attributes	
***Imaging Sites***	
Invicro, London,	455
Imperial IMANET PET centre, London	190
Wolfson Molecular Imaging Centre, Manchester	33
Seoul National University Bundang Hospital, Republic of Korea	114
***Controls** (%)*	195 (29.1)
***Age*** *year,* mean (min-max)	28.7 (18–65)
***Weight*** *kg,* mean (min-max)	75.9 (38–136)
***Gender*** *female (%)*	243 (33.9)
***Tomograph***	
Hi-Rez Biograph 6 (voxel size = 2.05 × 2.05 × 2 mm)	366
Biograph 40 Truepoint (voxel size = 1.59 × 1.59 × 1.5 mm)	113
Biograph TruePoint 6 (voxel size = 2.05 × 2.05 × 2 mm)	70
ECAT/EXACT3D (voxel size = 2.1 × 2.1 × 2.43 mm)	49
SIGNA PET/MR (voxel size = 2 × 2.05 × 2 mm)	19
***Injected Radioactivity*** *MBq,* mean (min-max)	188.2 (86.4–414.4)

All the scans were manually quality controlled in order to identify possible artifacts. The criteria used were: 1) plausible FDOPA PET signal distribution (identified by visual inspection), where the areas with highest PET uptake match anatomical regions with highest dopamine content, 2) max between frame motion realignment <8 mm (as derived by between-frame image realignment), 3) adequate anatomical atlas co-registration (identified by manually checking the striatal and cerebellar anatomical masks on individual FDOPA PET summed image).^46^

**Figure 2.**
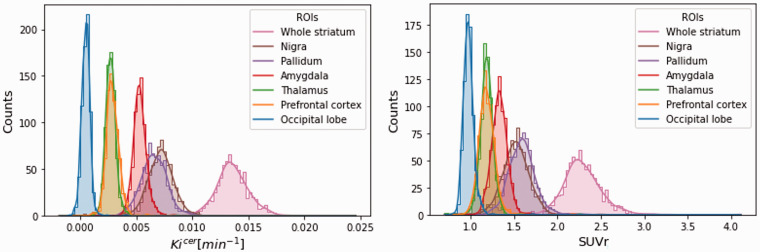
Distribution of Ki^cer^ and SUVr. Distribution of the Ki^cer^ (on the left) and SUVr (on the right) of the main region of interests (occipital lobe, prefrontal cortex, thalamus, amygdala, pallidum, substantia nigra, whole striatum) for all the subjects stored in XNAT.

The Ki^cer^ distributions across the brain are presented in Figure 2. Among the ROIs, the highest estimates were reported in the whole striatum *(Ki^cer^ mean ± SD: 0.0137* *±* *0.0015 min^−1^; min–max: 0.0102–0.0246 min^−1/^/SUVr mean±SD: 2.30 ± 0.22; min-max: 1.71–.11).* Outside the basal ganglia, the substantia nigra showed the highest signal *(Ki^cer^ mean ± SD: 0.0072 ± 0.0012 min^−1^; min–max: −0.0019–0.0122 min^−1^//SUVr mean ± SD: 1.53 ± 0.15; min–max: 0.82 2.49)* followed by the pallidum, the amygdala, the thalamus and the prefrontal cortex. The occipital lobe, which is sometimes used as reference region for FDOPA PET quantification instead of the cerebellum,^47^ showed the lowest estimates in ratio to the cerebellum *(Ki^cer^ mean±SD: 0.0005 ± 0.0004 min^−1^; min-max: −0.0009–0.0041 min^−1^//SUVr mean ± SD: 0.97 ± 0.06; min–max: 0.79–1.72).*

### Validation of the automated data analysis pipeline for FDOPA PET quantification

#### Comparison between automated and manual results

There was consistency between the XNAT and MATLAB pipeline, with a Ki^cer^ mean relative difference of 3.0 ± 5.5% and a Pearson’s correlation of 0.86. About 71% of the scans reported Ki^cer^ relative differences less than 5%.

**Figure 3.**
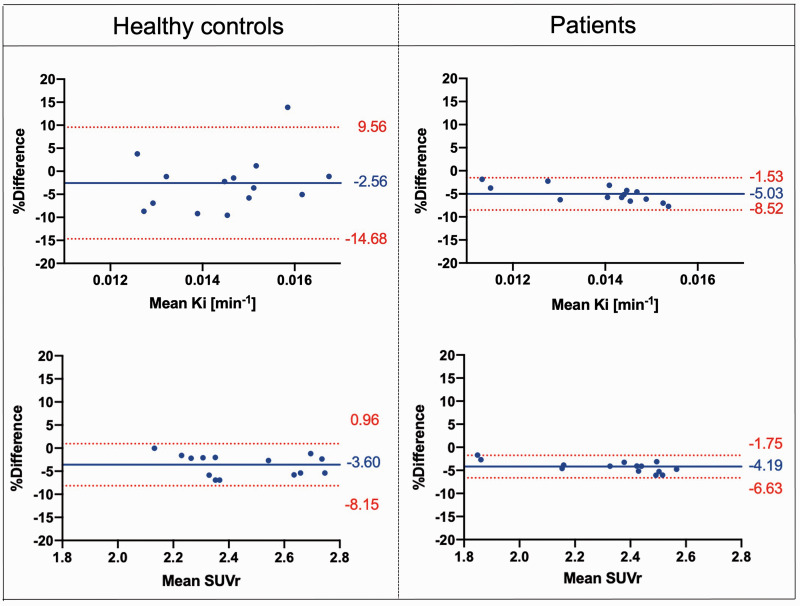
Bland-Altman. Plots comparing Ki^cer^ and SUVr estimates of the whole striatum with the XNAT-based and MATLAB-based pipelines, for both Dataset 1 and Dataset 2 [Difference (MATLAB – XNAT) vs. average]. The bias and 95% limits of agreement are reported in each graph.

In the test-retest datasets, the Bland-Altman plot confirmed good agreement between the XNAT and MATLAB pipelines for all the parameters, levels of analysis, datasets, and regions of interest. In the whole striatum, both XNAT-based Ki^cer^ and SUVr estimates are higher than the corresponding MATLAB ones within 5% mean relative difference (Figure 3). *We found a significant correlation between XNAT vs MATLAB relative differences and the magnitude of dopamine synthesis capacity estimates in patients, while the same correlation was not significant in the healthy control group. Given our relatively small sample size, any conclusions regarding the existence of true group differences in these correlations must be made with caution. In addition, using the Welch’s test we found a significant higher Ki^cer^ and SUVr variability in the healthy control group (p < 0.0001 and p = 0.0322, respectively), which could be possibly explain by the different technologies and performances of the PET tomographs used.* Consistently with the Bland-Altman analysis, XNAT-MATLAB Pearson’s correlation ranges from 0.64 to 0.99 for Ki^cer^, and from 0.79 to 1.00 for SUVr, with the lowest values for the limbic subvidision and the highest for the whole striatum/associative subdivision (Supplementary Table 2). The mean absolute difference between the two pipelines ranges from 3.4% to 9.4% for Ki^cer^, and from 2.5% to 12.4% for SUVr (Supplementary Table 2).

#### Assessment of test-retest reproducibility

**Figure 4.**
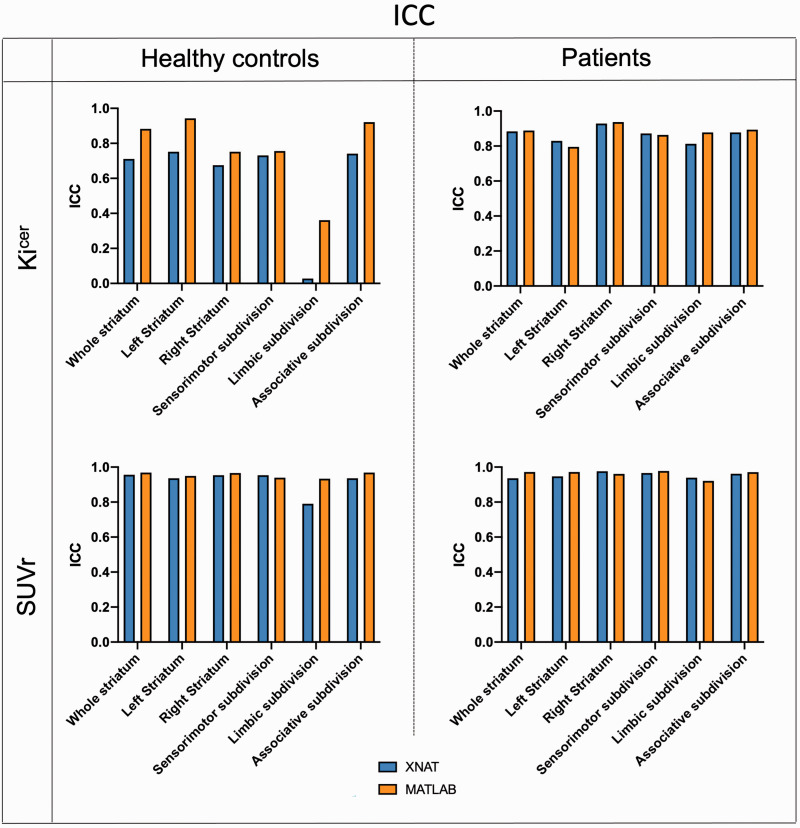
Test-retest intraclass correlation coefficient. Comparison of ICC between the XNAT (blue) and MATLAB (orange) pipelines for Ki^cer^ and SUVr of the whole striatum for both Dataset 1 and Dataset 2.

**Figure 5.**
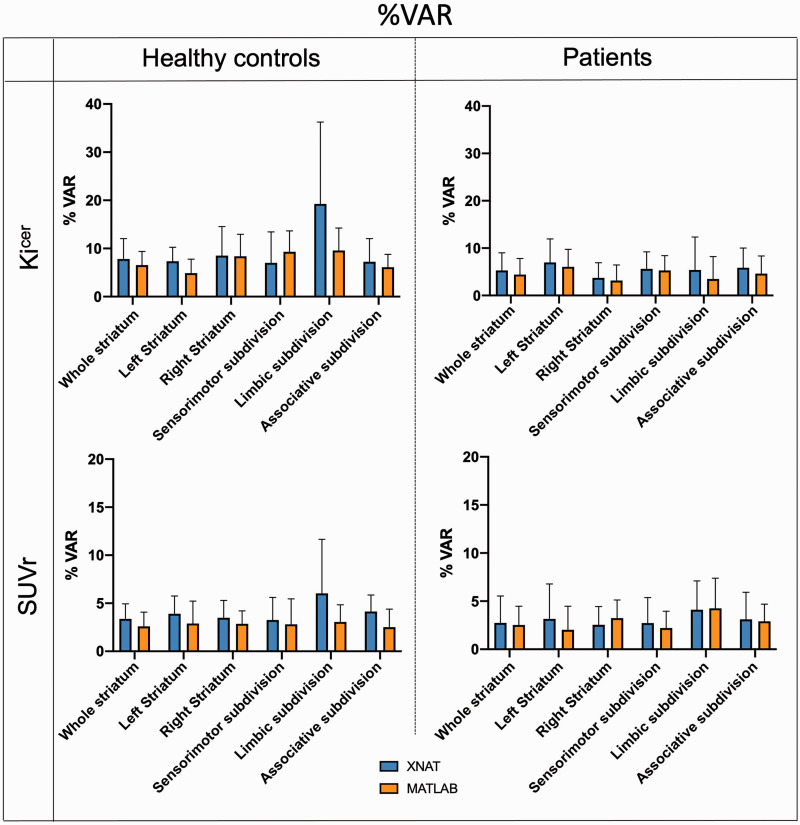
Test-retest mean percentage variability. Comparison of mean and 95% confidence intervals of %VAR between the XNAT (blue) and MATLAB (orange) pipelines for Ki^cer^ and SUVr of the whole striatum for both Dataset 1 and Dataset 2.

In terms of test-retest reliability and within-subject variation, the automated pipeline provided reproducibility and reliability of FDOPA PET in the striatum and its subdivisions (Figures 4 and 5) comparable to the ones previously described.^36^ Averaging the results from healthy controls and patients, the ICC for the Ki^cer^ estimates ranged from 0.421 for the limbic subdivision to 0.810 for the associative subdivision. The %VAR for the Ki^cer^ estimates ranged from 6.1 for the right striatum to 12.3 for the limbic subdivision. For the SUVr, the ICC ranged from 0.865 for the limbic subdivision to 0.965 for the right striatum, while %VAR ranged from 3.0 for the right striatum to 5.1 for the whole limbic subdivision.

#### Impact of motion-correction and atlas coregistration to kinetic modelling

Analysis of motion correction statistics highlighted that 6% of the scans (N = 46/792) had at least one motion spike, and 2.8% (N = 22/792) two or more spikes. The 85% of the total scans were patients. The mean and standard deviation of the motion detected for the healthy controls and patients (10.9 ± 6.6 mm and 13.8 ± 10.9 mm, respectively) were significantly different using the Mann-Whitney test.

There was a significant effect of the spikes on the Ki^cer^ estimates (*F = 8.84, p < 0.001*). Post-hoc tests showed that the Ki^cer^ significantly decreases for scans with two or three spikes (*p < 0.001*) (Supplementary Figure 4, graph on the left). Similarly, the SUVr significantly decreases for scans with five spikes (*p < 0.001*) (Supplementary Figure 4, graph on the right).

Analysis of the impact of atlas coregistration to kinetic modelling (Supplementary Table 3) showed that there is no difference in the Ki^cer^ estimates in the striatum and its subdivisions when using PETtoPET coregistration as compared with MRItoPET (striatal PETtoPET Ki^cer^ ICC = 0.884, striatal MRItoPET Ki^cer^ ICC= 0.831). On the other side, for the extrastriatal regions, MRItoPET coregistration signaficantly improved Ki^cer^ reprocibibility particularly in the globus pallidus (PETtoPET Ki^cer^ ICC = 0.259, MRItoPET Ki^cer^ ICC = 0.733), substantia nigra (PETtoPET Ki^cer^ ICC = 0.051, MRItoPET Ki^cer^ ICC = 0.825), amygdala (PETtoPET Ki^cer^ ICC = 0.402, MRItoPET Ki^cer^ ICC = 0.885) and anterior cingulate cortex (PETtoPET Ki^cer^ ICC = 0.609, MRItoPET Ki^cer^ ICC = 0.823). The %VAR for the Ki^cer^ estimates ranged from 5.4 for the hippocampus to 14.6 for the substantia nigra when using PETtoPET coregistration, and it ranged from 4.1 for the hippocampus to 13.2 for the thalamus when using the MRItoPET coregistration.

### Identification of demographical and experimental covariates for FDOPA PET imaging

There was a significant relationship between gender and striatal Ki^cer^ estimates *(F(1,109)=10.7, p < 0.001)*, with dopamine synthesis capacity being higher in women than men (marginal means, women Ki^cer^: 0.01353 ± 0.00147 min^−1^, men Ki^cer^: 0.01270 ± 0.00131 min^−1^, effect size = 2.056E-5). Age, weight, injected radioactivity, injected cold mass, specific activity and tomograph were not significantly associated with striatal Ki^cer^. A similar association between FDOPA PET and gender was also found when considering the subsample of scans (N = 103) acquired only using the Siemens Biograph tomograph only *(F(1,98) = 10, p = 0.002,* women Ki^cer^: 0.01354 ± 0.00145 min^−1^, men Ki^cer^: 0.01268 ± 0.00130 min^−1^) (Table 2). In contrast, none of the experimental and demographic variables were significantly associated with SUVr (Table 2).

**Table 2.** Association of demographical and experimental variables with Kicer and SUVr of whole striatum region.

Dependant variable: Whole striatum Ki^cer^	Full sample (n = 115)			With Siemens biograph only (n = 103)		
	Pearson r	T	p	Pearson r	t	p
*Age*	−0.082	−1.448	0.151	−0.143	−1.449	0.151
*Weight*	0.002	1.015	0.312	−0.028	0.960	0.339
*Gender*	−0.289	−3.267	<0.001*	−0.299	−3.162	0.002*
*Injected radioactivity*	0.025	0.005	0.996	0.024	0.053	0.958
*Tomograph*	0.059	1.028	0.306	0.053	0.796	0.428
	Full sample (n = 115)			With Siemens biograph only (n = 103)		
Dependant variable: Whole striatum SUVr	Pearson r	T	p	Pearson r	t	p
*Age*	−0.116	−1.072	0.286	−0.040	−0.206	0.837
*Weight*	0.011	0.679	0.498	0.017	0.575	0.567
*Gender*	−0.094	−1.102	0.273	−0.108	−1.166	0.246
*Injected radioactivity*	0.003	0.303	0.762	0.126	1.383	0.170
*Tomograph*	−0.057	−0.117	0.907	0.039	0.768	0.444

Significance is indicated with asterisks.

## Discussion

In this study we characterized a standardised infrastructure for FDOPA PET neuroimaging to quantify brain dopamine function in living human brains. The platform was built from a harmonized FDOPA repository, integrating clinical and demographic information, together with FDOPA PET data. To our knowledge, this is the largest dataset of this type to date, including hundreds of scans with a very comparable acquisition protocol. The automated analysis framework for FDOPA PET quantification, directly embedded in the platform, ensured full control on the analytical process and replicable results on the stored data, confirming the good replicability and reproducibility of the FDOPA PET measures in the striatum and its subdivisions.

### Data harmonisation

All the FDOPA PET imaging data were acquired using similar methods, which facilitated their integration in a unique storage infrastructure. However, the collection of FDOPA PET data from multicenter studies and acquired with multi generation of PET scanners with more 20 years of technological differences inevitably led to inconsistencies in the data structure and format used. In our cases most of the issues were linked to the different time point of data acquisition and scanner manufactures used, which led to different image formats (e.g. dicom, analyze, nifty, ecat v7), different data decay correction and attenuation correction and different voxel size. To overcome these differences, the data were renamed using the same file name convention and manually converted to the same format prior uploading them in the XNAT platform, following same PET imaging standard as implemented in MIAKAT (e.g.neurological convention, 2 mm isotropic voxelsize, data tracer decay corrected).^48^ A rigid name convention was also used for the processed data obtained from the analysis pipeline, to ensure traceability across the entire dataset. This solution has been bespoken for the particular case study; however other name conventions might have been equally effective. The brain PET imaging community has lacked data format standards, and only very recently it has been recognised the need of more standardized data structure.^49,50^ The hope is that initiatives like PET-BIDS will address the heterogeneity of data organization by following the FAIR principles (findability, accessibility, interoperability, and reusability).^51^

### Validation of the automated analysis framework for FDOPA PET quantification

Consistently with our research objectives, the automated pipeline of analysis demonstrated to provide robust and replicable results consistent with current stardard of FDOPA brain PET data analyisis.

The percentage difference in the Ki^cer^ quantification between the manual and automated pipeline was within the acceptable threshold of 10% for 94% of the data. The percentage difference can be partially explained by the intrinsic complexity of the neuroimaging data analysis.^52^ In this study, the XNAT-based pipeline was implemented from the available MATLAB code, but reproducing the exact results when using different analytical pipelines can be quite challenging due to the lack of standardized analytical pipelines and the lack of complete description of the used methodologies.^26^ The programming frameworks used, the computer environment and the choice of the pre-processing strategies are just few of the possible reasons behind different analytical outcomes. The discrepancies in the FDOPA quantification between the MATLAB and XNAT pipelines found in this study might come primarily from the pre-processing steps, which include motion correction, atlas coregistration and noise filtering. It is well-known that pre-processing steps are a critical part of a PET analysis framework, and small differences can impact the results.^53^ In support of this aspect, we confirmed that the same Ki^cer^ and SUVr estimates were obtained when MATLAB-based preprocessed data were given in input to the XNAT-based pipeline (results not shown). It is also relevant to note that the two pipelines use different programming languages, MATLAB and Python, and different software packages, introducing another source of discrepancies in the data quantification. Unfortunately these are third-party components that are difficult to be controlled for. Hence the importance of keeping track of the software, libraries and packages used, as well as of all the steps used in the analysis framework, to ensure replicability and reproducibility.

The FDOPA Ki^cer^ and SUVr measures obtained with the XNAT pipeline have reproducibility and reliability in the striatum and its subdivisions comparable to the ones presented in Egerton et al in terms of test-retest variability and within-subject variation,^36^ except for the limbic subdivision. The limbic subdivision showed in fact high Ki^cer^ variability and low ICC with the XNAT pipeline compared to the MATLAB for the healthy controls. These discrepancies in the Ki^cer^ quantification could be in part explained by the image pre-processing (motion correction, segmentation and coregistration). The limbic subdivision is a small region, more susceptible to motion artefacts and partial volume effects, and this can affect the amount of activity measured.^54^ The reproducibility and replicability of dopamine synthesis capacity estimates in the striatum and its subdivisions were also insensitive to the type of atlas coregistration method used. This was not the case for the extrastriatal regions, for which only the use of MRI mediated coregistration allowed to obtained statistically reproducible estimates. This is well-known in PET imaging analysis, for which structural MRI are typically used to facilitate the tissue brain segmentation, otherwise difficult when using only PET-based imaging data.^54^

### Technical and biological factors impacting FDOPA PET quantification

The availability of the proposed dataset allowed to characterise the distribution of the FDOPA PET signal across regions. The Ki^cer^ estimates in the whole striatum are the highest, ranging from 0.0102 to 0.0246 min^−1^ across individuals. In contrast to some recently published studies,^55,56^ Ki^cer^ estimates smaller than 0.010 min^−1^ were only detected in scans with high head motion parameters, and rejected as outliers. Given the association between motion parameters and dopamine synthesis estimates, it becomes extremely important to physically limit head movements during the image acquisition. However, this can be uncomfortable and practically challenging. Alternative strategies to control and correct for data with high motion might be necessary, expecially in long acquisition. Usually the approach used to detect and exclude data affected by motion is study/site dependent and this can introduce further discrepancies in the results, which become hardly comparable. In addition, motion in patients and controls are different and this could have an effect in cross sectional studies. It is important to note that we assumed our data to have satisfactory alignment between transmission (CT or MRI) and emission scans. Before any type of analysis, all the PET scans had to pass a quality control step where we looked for the presence of image artefacts due to misregistration of PET data with transmission scans. In case of major issues, individual PET frames were realigned to the attenuation scans offline and re-input into the scanner for new reconstruction. Moreover, our protocol allowed to reacquire a second attenuation scan when the acquisition was interrupted halfway through (i.e. the participant needs to go to the toilet).

The type of analysis pipeline is not the only factor that leads to FDOPA PET differences between published studies: in Eisenberg et al,^56^ for example, the study participants did not receive entacapone prior imaging acquisition, which is known to have a significant effect on FDOPA PET quantification both in animal^57^ and humans.^58,59^ It would be interesting to analyse FDOPA PET imaging without pre-administration of entacapone but unfortunately these data are not available as part of our repository. However, it is worth noting that differences in the acquisition protocol would not impact the proposed analytical framework, even if differences in signal-to-noise ratio across protocols could affect the comparability of the estimates of dopamine synthesis capacity obtained with other protocols. Differences in timing of acquisition^60^ are an additional source of variability to consider: kinetic parameters quantified by shorter PET acquisitions (e.g. 60 minutes) do not return the same estimates.^20,61^ Intermittent rather than continuous acquisition have also been proposed, but without a direct comparison with standard approaches the interpretation remains difficult.^60^

A standardized protocol of data analysis allows to reduce variability^61^ across studies. For example, the use of apparently “innocent” different experimental settings or analytical options, such as the use of different atlases for defining the reference region can introduce variability, which can be problematic if collinear with the effects of interest, i.e. different impact on estimates obtained from patients and controls (Supplementary Table 4). Taken together, these factors highlight the importance of using a standardised acquisition protocol as well as common data analysis platform to compare results across studies.

*The finding of a gender effect on the FDOPA measures, with higher Ki^cer^ and SUVr in female than men, is in agreement with established gender differences in brain dopaminergic activity.* ^62^ *^,^* ^63^ *Such differences might be explained by the effect of gonadal hormones, which modulate behavioral and neurochemical indices of activity in the striatum. Recent rodents studies have shown that, in female rats, estrogen increases presynaptic dopaminergic activity,* ^64^ *while a higher density of the striatal dopamine transporter is found in male rats^.^* ^65^ *^,^* ^66^ *If such findings translate to humans, they may explain some of the differences found in our study. Recently both higher diffuse cortical and lower subcortical FDOPA uptake were found in women compared to man.* ^67^ *The authors linked these differenes to multiple factors including cerebral blood flow and effects of estrogen to cerebral metabolism.* ^67^ *Despite gender is an important biological variable for different mental disorders, its influence on FDOPA uptake remains poorly known. Having a more comprehensive understanding on how it affects dopamine function would be of particular interest for the future development of individualized treatment response algorithms.* ^68^

### Limitations

In this study the data were manually quality controlled by visually inspecting the raw FDOPA tracer time-activity course, and the Ki^cer^ estimates and motion parameters obtained from the FDOPA quantification pipeline. This type of analysis is vulnerable to inconsistencies due to between-operator differences. Automatic pipelines for data quality control tailored to investigate specific characteristics of the data collected and possible mis-alignement of the data from a reference space would reduce such issues,^46^ but validated solutions are still missing.

Data provenance, defined as the documentation of where the data comes from and the processes and methodology by which it was produced, is fundamental to ensure full reproducible experimental and analytical processes.^69^ The pipeline for FDOPA PET quantification, embedded in the platform, will need to be supported by documentation of the whole analytical process to further support and ensure full reproducibility of the scientific results. In terms of harmonization, the data were renamed using the same file name convention and manually converted to the same format to guarantee homogeneity across the database. However, the future aim is to use a unique standardized data format and structure, such as PET-BIDS, which would facilitate the integration of data from different sources and sites and ensure a more reliable data harmonization.

The analysis pipeline implemented in this work follows the pipeline described in the variety of FDOPA imaging studies published by the Psychiatric Imaging Group (King’s College London). The pipeline could be further improved by integrating information from structural data like T1w MRI, which were not yet available for all the FDOPA PET scans, which could be used to enhance the atlas coregistration step, as shown for the patient test-retest dataset. However, this study is only based on PET coregistration, since additional MRI data were available only for a restricted number of FDOPA PET scans.

XNAT was chosen as the platform for the implementation of the proposed infrastructure and no other alternatives were considered. XNAT was established in 2006 and since then it has been extensively used by research groups to host and collect clinical and other data associated with the initial raw imaging studies, enabling a broad range of collaborative research.^70^ The system can be easily extended to other applications and new XNAT instances can be tailored to the different neuroimaging biomarkers, and the corresponding analytical methods can be integrated as automatic pipelines. However, XNAT is strongly imaging-driven and struggles to capture the variety and complexity of non-imaging data that are often acquired in modern experimental medicine studies. This information is important and could be integrated with neuroimaging data to provide a deeper individual phenotyping. The integration of this information in a unique system would permit to create a patient-centric platform, moving towards precision medicine.

In terms of analysis of the demographical and experimental covariates, only gender showed a significant effect on FDOPA measures. Whether this is a true effect or rather reflects differences in tracer metabolism or intravascular activity between genders, it is something our data cannot yet fully explain. Future studies attempting to replicate this finding and including the measurement of metabolites in both plasma and brain tissue would be important to clarify whether the effect of gender we report here is due to differences in systemic rather than central dopamine synthesis and metabolism. In addition, Participants included in the dataset had a limited rage of age variation [18–65 years], with the majority being situated between 18 and 30 years old. Including data from older cohorts will be a necessary step to more robustly explore a possible association between age and the FDOPA PET signal.

In addition, we modelled the effect of PET tomograph as covariate to account for the cumulative effect of differences among datasets in aspects of the PET physics (e.g. reconstruction, attenuation correction, sensitivity of scanners, etc), without exploring the effect of each factor separately.

## Conclusions

This study presents an automatic pipeline for FDOPA PET quantification, which has been validated on a unique harmonized FDOPA PET repository. The availability of such large FDOPA PET repository has permitted to investigate the reproducibility and reliability of the analytical method, as well as to study the effect of processing parameters, demographic and experimental variables on the FDOPA quantification. The proposed robust analytical framework aims to facilitate FDOPA PET imaging implementation across sites and research institutions and to boost the use of FDOPA PET as a clinical biomarker in psychosis and other mental disorders.

## Supplemental Material

sj-pdf-1-jcb-10.1177_0271678X231168687 - Supplemental material for An automatic analysis framework for FDOPA PET neuroimaging Click here for additional data file. Supplemental material, sj-pdf-1-jcb-10.1177_0271678X231168687 for An automatic analysis framework for FDOPA PET neuroimaging by Giovanna Nordio, Rubaida Easmin, Alessio Giacomel, Ottavia Dipasquale, Daniel Martins, Steven Williams, Federico Turkheimer, Oliver Howes, Mattia Veronese, and the FDOPA PET imaging working group:Sameer Jauhar, Maria Rogdaki, Robert McCutcheon, Stephen Kaar, Luke Vano, Grazia Rutigliano, Ilinca Angelescu, Faith Borgan, Enrico D’Ambrosio, Tarik Dahoun, Euitae Kim, Seoyoung Kim, Micheal Bloomfield, Alice Egerton, Arsime Demjaha, Ilaria Bonoldi, Chiara Nosarti, James Maccabe, Philip McGuire, Julian Matthews and Peter S Talbot in Journal of Cerebral Blood Flow & Metabolism

## Data Availability

The data that support the findings of this study are available from The NeurOimaging DatabasE (NODE) repository (https://maudsleybrc.nihr.ac.uk/research/precision-psychiatry/neuroimaging/neuroimaging-database-node/) but restrictions apply to the availability of these data, which were used under license for the current study, and so are not publicly available. Data are however available from the authors upon reasonable request and with permission by the data controller institutions, by contacting the support team (node.information@kcl.ac.uk) or the author Dr. Giovanna Nordio (giovanna.nordio@kcl.ac.uk).

## Software availability statement

The software has been documented and stored in our GitHub private repository, and access can be granted upon reasonable request.
