# Supplementary material for: An automatic analysis framework for FDOPA PET neuroimaging
Source: J Cereb Blood Flow Metab. 2023 Apr 7;43(8):1285–300. doi: 10.1177/0271678X231168687 (PMC10369152; doi:10.1177/0271678X231168687)
Supplement: sj-pdf-1-jcb-10.1177_0271678X231168687 - Supplemental material for An automatic analysis framework for FDOPA PET neuroimaging [file sj-pdf-1-jcb-10.1177_0271678X231168687.pdf]

An automatic analysis framework for FDOPA PET neuroimaging

Nordio Giovanna et al

Supplementary Figures

Supplementary Figure 1: Schematic flowchart of the proposed infrastructure. The data are collected (1) and stored in XNAT (2). The main analysis pipeline for FDOPA PET quantification (3) and the summary pipeline (4) run automatically on the data, and the output are stored following the same data structure.

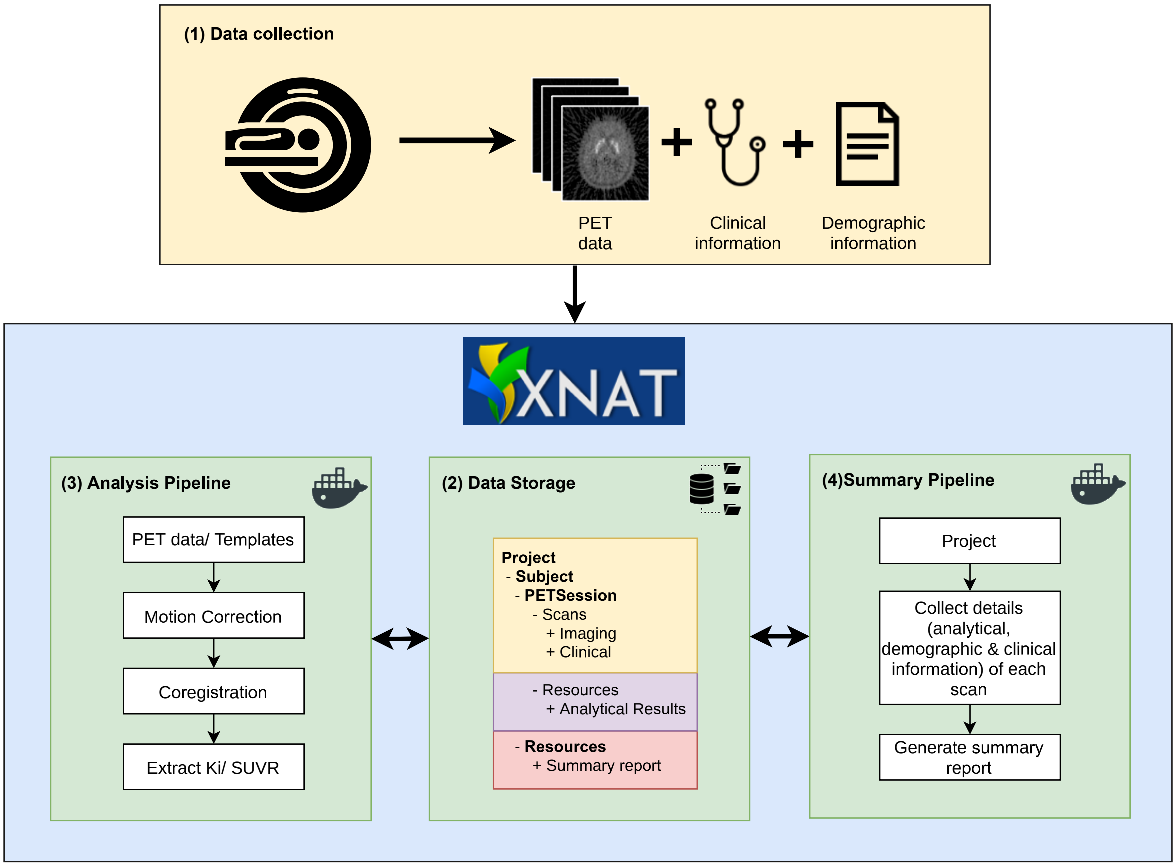

An automatic analysis framework for FDOPA PET neuroimaging

Nordio Giovanna et al

Supplementary Figure 2: Single frame FDOPA PET imaging of a representative subject. A representative brain slice (axial view) is reported for each of the 32 frame collected. The reference frame at 15 minutes used in the motion correction step is indicated in red.

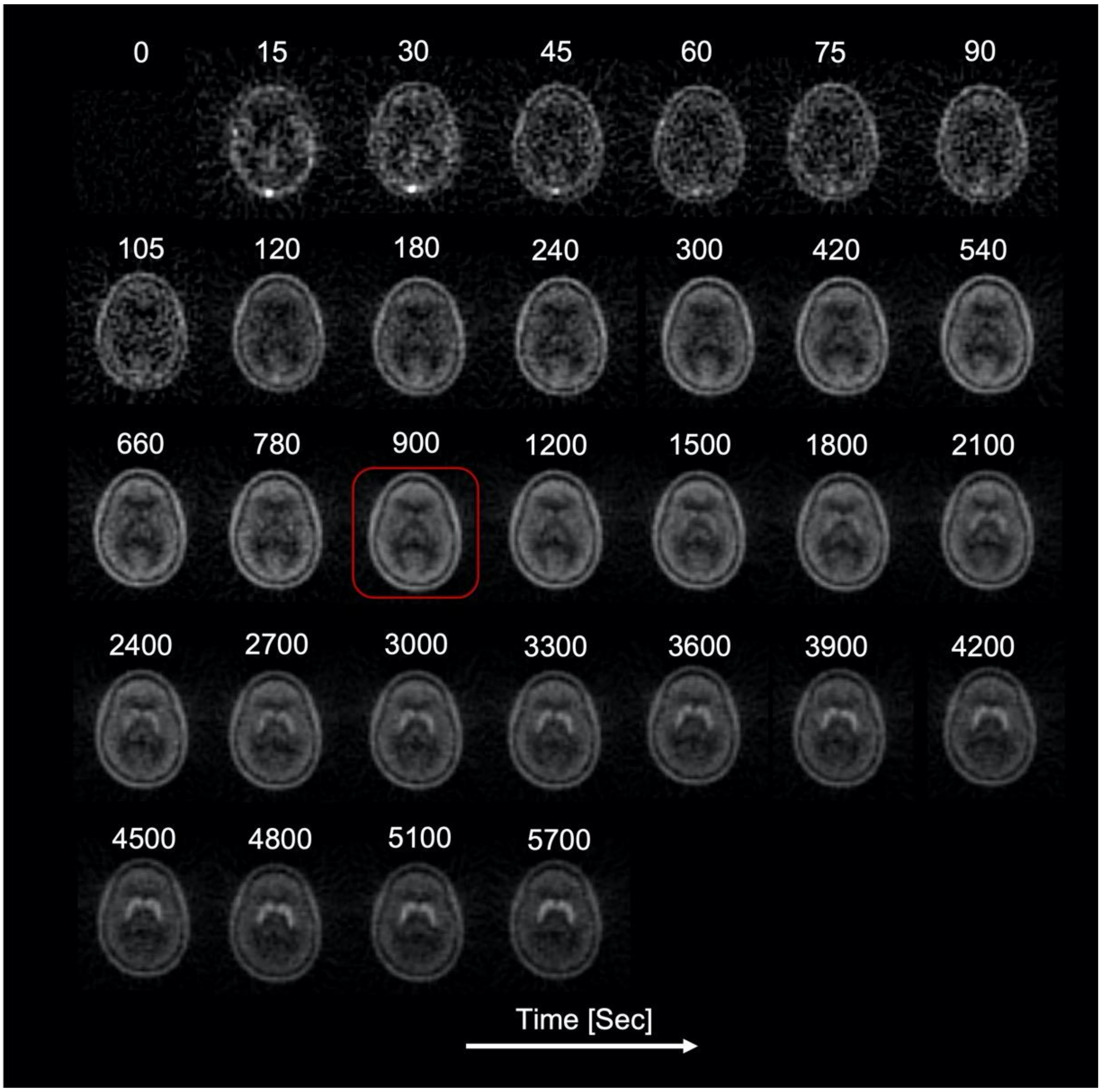

An automatic analysis framework for FDOPA PET neuroimaging

Nordio Giovanna et al

Supplementary figure 3: Anatomical brain segmentation. Striatum and its subdivisions (top panel), pallidum, cerebellum and nigra region (bottom panels) overlaid on a grayscale anatomical T1-weighetd MRI template in MNI standard space.

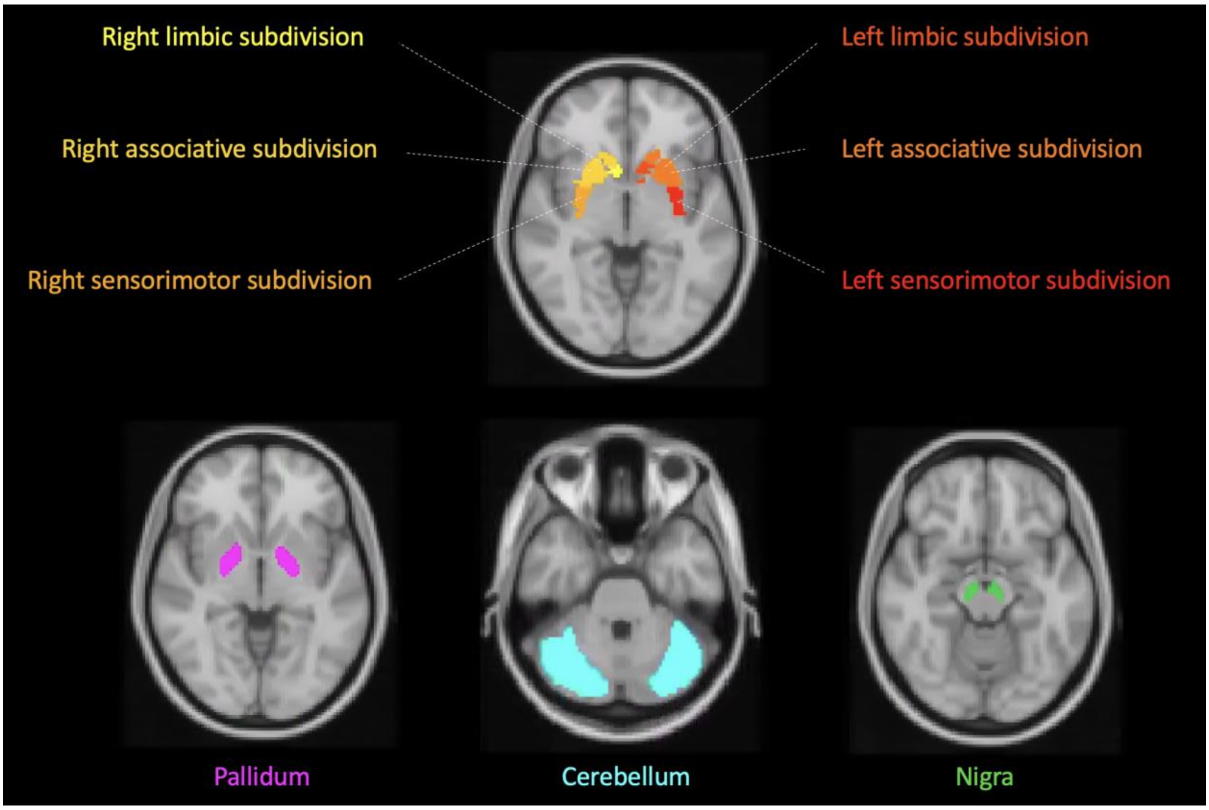

An automatic analysis framework for FDOPA PET neuroimaging

Nordio Giovanna et al

Supplementary Figure 4: FDOPA quantification and spikes. Mean and 95% confidence intervals of the Ki^{cer} (A) and SUVr (B) of the whole striatum as a function of the number of spikes detected during the motion correction step of the analysis pipeline.

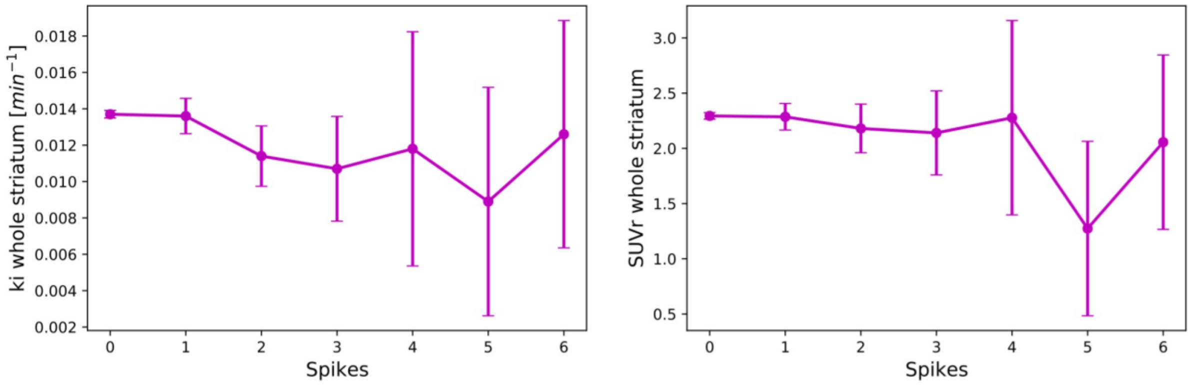

An automatic analysis framework for FDOPA PET neuroimaging

Nordio Giovanna et al

Supplementary Tables

Supplementary Table 1: Overview of the software tools and packages used in the study

Software & packages	Description
**XNAT**	Extensible open-source imaging informatics software platform dedicated to imaging-based research
**Representational State Transfer (REST) Application-Program Interface (API)**	Application programming interface that follows the constraints of REST architecture
**Container Service plugin**	XNAT plugin for controlling containers
**Docker container image**	Lightweight, standalone, executable package of software that includes everything needed to run an application
**SPM 12**	Statistical parametric mapping software
**NumPy**	Numerical Python library
**NiBabel**	Neuroimaging Python library
**SciPy**	Scientific and technical computing Python library
**Jamovi**	Open statistical software for the desktop and cloud

Supplementary Table 2: ***Comparison of dopamine synthesis capacity estimates.*** *The table reports XNAT-based Ki^{cer} and SUVr estimates (for controls and patients, respectively) and a comparison with MATLAB-based estimates.".*

Ki^{cer}	Healthy controls				Patients			
	XNAT estimates [min^{-1}]	MATLAB estimates	Pearson's correlation r	Mean absolute difference [%]	XNAT estimates	MATLAB estimates [min^{-1}]	Pearson's correlation r	Mean absolute difference [%]
Whole striatum	0.0152±0.0012	0.0144±0.0015	0.79	8.2	0.0143±0.0015	0.0136±0.0012	0.99	5.1
Left striatum	0.0154±0.0013	0.0141±0.0016	0.77	8.4	0.0142±0.0017	0.0139±0.0012	0.96	3.4
Right striatum	0.0150±0.0013	0.0147±0.0015	0.79	8.4	0.0145±0.0013	0.0133±0.0013	0.97	8.7
Sensorimotor subdivision	0.0159±0.0015	0.0156±0.0018	0.89	8.6	0.0151±0.0014	0.0139±0.0011	0.97	8.5
Limbic subdivision	0.0125±0.0009	0.0150±0.0009	0.64	9.3	0.0122±0.0011	0.0130±0.0012	0.84	8.3
Associative subdivision	0.0155±0.0013	0.0137±0.0016	0.79	7.0	0.0145±0.0017	0.0136±0.0013	0.99	6.2

SUVr	Healthy controls				Patients			
	XNAT estimates	MATLAB estimates	Pearson's correlation r	Mean absolute difference [%]	XNAT estimates	MATLAB estimates	Pearson's correlation r	Mean absolute difference [%]
Whole striatum	2.52±0.21	2.40±0.18	0.93	4.0	2.37±0.26	2.27±0.23	1.00	4.3
Left striatum	2.55±0.19	2.42±0.17	0.93	5.1	2.35±0.27	2.32±0.23	0.97	2.5
Right striatum	2.49±0.22	2.39±0.19	0.92	3.3	2.40±0.25	2.22±0.23	0.95	8.0
Sensorimotor subdivision	2.67±0.23	2.63±0.19	0.93	2.8	2.57±0.28	2.39±0.23	0.99	7.1
Limbic subdivision	2.15±0.17	2.42±0.15	0.79	12.4	2.10±0.19	2.18±0.22	0.94	5.4
Associative subdivision	2.52±0.22	2.31±0.18	0.93	8.0	2.35±0.27	2.24±0.24	0.99	5.1

Supplementary Table 3: *Reproducibility (ICC and %VAR) of the Ki^{cer} estimates in the striatum and its subdivisions and five extra-striatal regions when using PET and MRI data for the atlas coregistration. For this analysis, the test-retest dataset of patients with psychosis was used.*

Ki^{cer}	Coregistration with PET data		Coregistration with MRI data	
	%VAR	ICC	%VAR	ICC
STRIATAL REGION AND SUBDIVISIONS				
Whole striatum	5.3	0.884	7.5	0.831
Left striatum	7	0.830	9.6	0.756
Right striatum	3.7	0.929	6.4	0.871
Sensorimotor subdivision	5.6	0.872	8.9	0.406
Limbic subdivision	5.4	0.813	10	0.655
Associative subdivision	5.9	0.878	6.6	0.916
EXTRASTRIATAL REGIONS				
Globus pallidum	9.8	0.259	10.3	0.733
Substantia nigra	14.6	0.051	11.7	0.825
Thalamus	10.9	0.787	13.2	0.689
Hippocampus	9.2	0.629	13.0	-0.499
Amygdala	5.4	0.402	4.1	0.885
Anterior Cingulate Cortex	12.1	0.609	9.1	0.823

Supplementary Table 4*: Comparison of Ki^{cer} estimates when defining the cerebellum region extract using the Hammersmith atlas as opposed to the Martinez atlas (all the other analytical settings remain the same). Table summarizes the mean correlation and the mean percentage difference, separating by group and region-of-interest.*

Ki^{cer}	Healthy controls		Patients	
	**Correlation**	**Mean absolute difference**	**Correlation**	**Mean absolute difference**
Whole striatum	0.87	4.4	0.97	5.2
Left striatum	0.87	4.2	0.97	5.8
Right striatum	0.89	4.7	0.95	4.7
Sensorimotor subdivision	0.85	5.0	0.91	7.1
Limbic subdivision	0.94	5.5	0.67	9.7
Associative subdivision	0.87	4.7	0.97	5.1
